# A Hybrid Optimization Method for Solving Bayesian Inverse Problems under Uncertainty

**DOI:** 10.1371/journal.pone.0132418

**Published:** 2015-08-07

**Authors:** Kai Zhang, Zengfei Wang, Liming Zhang, Jun Yao, Xia Yan

**Affiliations:** 1 China University of Petroleum, 66 Changjiang West Road, Qingdao, Shandong, 266555, China; 2 PetroChina Coalbed Methane Company Limited, Beijing, 100028, China; Beijing University of Posts and Telecommunications, CHINA

## Abstract

In this paper, we investigate the application of a new method, the Finite Difference and Stochastic Gradient (Hybrid method), for history matching in reservoir models. History matching is one of the processes of solving an inverse problem by calibrating reservoir models to dynamic behaviour of the reservoir in which an objective function is formulated based on a Bayesian approach for optimization. The goal of history matching is to identify the minimum value of an objective function that expresses the misfit between the predicted and measured data of a reservoir. To address the optimization problem, we present a novel application using a combination of the stochastic gradient and finite difference methods for solving inverse problems. The optimization is constrained by a linear equation that contains the reservoir parameters. We reformulate the reservoir model’s parameters and dynamic data by operating the objective function, the approximate gradient of which can guarantee convergence. At each iteration step, we obtain the relatively ‘important’ elements of the gradient, which are subsequently substituted by the values from the Finite Difference method through comparing the magnitude of the components of the stochastic gradient, which forms a new gradient, and we subsequently iterate with the new gradient. Through the application of the Hybrid method, we efficiently and accurately optimize the objective function. We present a number numerical simulations in this paper that show that the method is accurate and computationally efficient.

## Introduction

**Fig 1:**
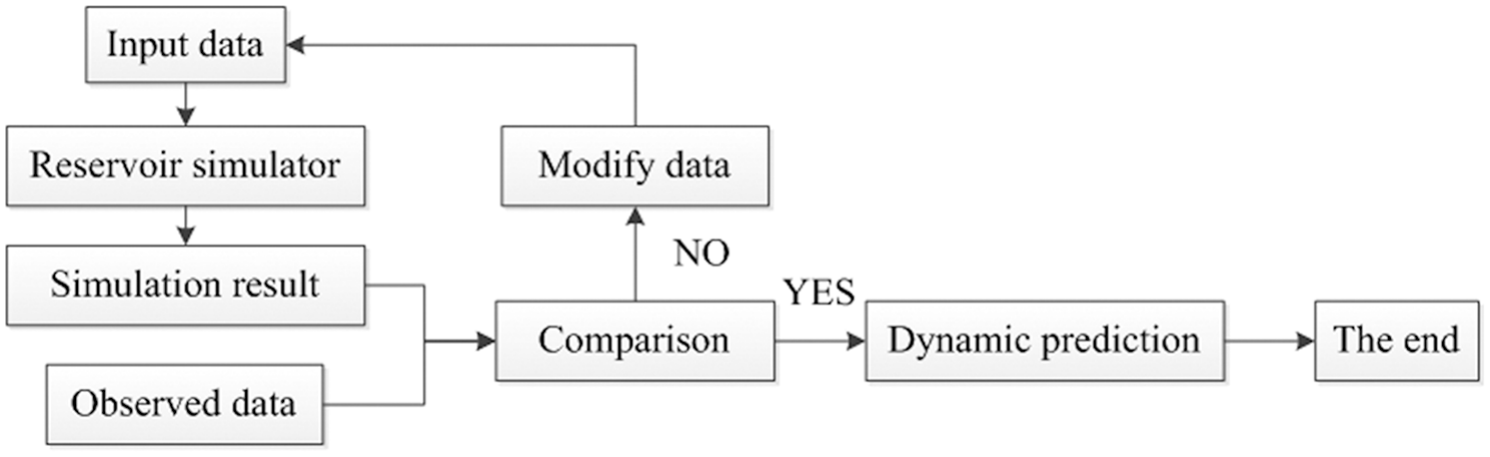
The process of history matching. Input data are the parameters generated based on the probability distribution. Observed data are the production data measured on site.

Subsurface geology is always uncertain. Uncertainty assessment of geological description and reservoir production prediction is an inverse problem and is usually performed by generating a suite of plausible realizations of the reservoir model that are consistent with the available data (Fig 1). Randomized maximum likelihood (RML) was introduced by Oliver [1] and is used to build an a posteriori probability density function (PDF). Generating a realization with RML involves minimizing an objective function with an optimization method [2, 3]. In the early studies of history matching, the least square method was used for optimization. Because the objective function usually contains a large number of parameters, the descending dimension method was introduced into the optimization process to reduce the computation time.

There are two different types of methods for optimization in history matching: the gradient-based method and the randomized method. The gradient obtained from the gradient-based method can be calculated by either using the Jacobi or adjoint method. Due to the complexity of solving the Hessian matrix, which is required in the gradient-based method, it could take many iteration steps to obtain a small value of the objective function. The Quasi-Newton method, which does not require calculating the Hessian matrix, was used by P. Yang [4] to minimize the objective function; however, due to having a long computation time, it can only be used in one- or two-dimensional flows. The Gauss-Newton method was later applied to simulate three-phase flows and extend the scope of the history matching, even though the computation of the gradients is often more expensive than solving the flow equations. To overcome this problem, some have used fast streamline-based simulation methods for history matching [5–7].

The randomized methods have overcome some of the drawbacks of gradient-based methods because there is no need to calculate the accurate gradient; instead, one only obtains the stochastic gradient by the value of the objective function. Simulated Annealing and Evolutionary Algorithms such as genetic algorithms [8] and Evolution Strategies have been adapted in various reservoir performance optimization frameworks, Ant colony foraging optimization algorithm [9, 10]which is drived from ant colony foraging process is one of global search methods. The ant will leave a kind of hormone in the process of foraging and the subsequent ants can identify the amount of these hormones to judge whether this path is the best route. Through this algorithm we can get the optimal solution. What they have in common is that a large amount of calculation and a slow rate of convergence. For large practical optimization instances, they have a narrow applicability because the computation time is long. For this reason, the Ensemble Kalman filter method was introduced into the reservoir history matching field. The Ensemble Kalman filter (EnKF) is one of the Monte Carlo approaches for history matching [11, 12]. The EnKF method has resolved many intractable reservoir problems in recent years.

The simultaneous perturbation stochastic approximation (SPSA) algorithm, which was developed on the basis of the Kiefer-Wolfowitz stochastic approximation algorithm [13], has also attracted much attention for challenging optimization problems in which it is not easy to directly obtain a accurate gradient of the objective function for the variavles being optimized [13, 14]. SPSA is an easily implemented and highly efficient stochastic gradient that relies on an objective function. In contrast, the finite difference approach requires a lot of function measurements for which the amount is proportional to the dimension of the gradient vector. Applications of the SPSA include training neural networks, monitoring signals, et al. H. Klie [15] and Gao [16] introduced SPSA into the reservoir history matching field. Without considering the correlation of the model parameters, SPSA reaches an unsatisfactory goal. For this reason, Li and Reynolds [17] replaced the Bernoulli distribution with a Gaussian distribution of disturbance variables and introduced a covariance matrix into SPSA; this approach has a better matching result but has a slow rate of convergence.

At present, those methods which combine many different kinds of optimization algorithms have been widely applied in the reservoir production optimization field [18, 19]. We adopt an approach by combining partial finite differences with the stochastic method based on a directional derivative to solve the inverse problem and quantify the difference frequency in order to increase the stability of this method. This method (called the Hybrid method) mainly replaces the corresponding important components of the stochastic gradient in sequence and integrates them to create a new approximation gradient for optimization. Examples of using the Bayesian approach to solve engineering problems can be found in [19–26] When quantifying the uncertainty in the model parameter identification problems, the novelty of the present work rests in the demonstration of the potential for accelerating the solving time of inverse solution. By employing the Hybrid method and the improved algorithm in the inverse solution process, the solution time can be considerably reduced.

## Problem Formulation

### Objective function

The Randomized Maximum Likelihood Estimation (RMLE) is one of the methods that can be performed to find the value of *x* by Probability theory, where the overall probability function *p*(*X*,*θ*) is known, and *θ* is an unknown parameter of the set Ф.

We filter *N*_*e*_ random samples, which are defined as $X_{1},X_{2},\cdots ,X_{N_{e}}$, from the population *X*, and obtain the corresponding actual observed values $x_{1},x_{2},\cdots ,x_{N_{e}}$. Then, the probability that *X*_1_ = *x*_1_, $X_{2}=x_{2},\cdots ,X_{N_{e}}=x_{N_{e}}$ occurs simultaneously is

$$p(\theta )=\prod_{i=1}^{N_{e}}p(X_{i}=x_{i})$$ (1)

Due to Probability theory, the probability function *p*(*θ*) can obtain the maximum value.

In engineering problems, parameters (such as porosity, permeability) usually have a type of probability distribution. From experience, we know that these parameters usually obey a Gaussian distribution in an actual application.

The Probability Density Function is

$$p(x)=\frac{1}{\sqrt{2\pi }\sigma }e^{-\frac{{\left(x-\mu \right)}^{2}}{2\sigma^{2}}}$$ (2)

Where *x* ~ *N*(*μ*,*σ* ^2^), *μ* is the mean of *x*, and σ^2^ is the variance.

Eq 2 can also be expressed as

$$p\left(x\right)\propto \text{exp}\left[-\frac{1}{2}{\left(x-x_{av}\right)}^{T}C_{X}^{-1}\left(x-x_{av}\right)\right]\;x\in N\left(x_{av},C_{X}\right)$$ (3)

Where *x* denotes the *N*_*e*_ dimensional vector, which includes uncertain parameters; and *x*_*av*_ denotes the prior model parameters; *C*_*X*_ is the *N*_*e*_ × *N*_*m*_ covariance matrix of the measurement errors that are generated by the statistical methods, and $C_{X}^{}\in R^{N_{e}\times N_{m}}$.

Based on statistical theory, the relationship between the observed production data and the reservoir model parameters in the oilfield development process is

$$d_{\text{obs}}=g\left(x\right)+\varepsilon_{r}\;\varepsilon_{r}\in N\left(0,C_{D}\right)$$ (4)

Where *d*_obs_ denotes the *N*_d_ dimensional vector that includes the actual observed data; *g* represents the numerical simulator of the reservoir system; *ε*_*r*_ is the measurement error; and *C*_*D*_ is the covariance matrix.

Maximum likelihood estimation can be performed to find the parameters that maximize the probability that the calculated value is the same as the observed value; thus, the conditional probability distribution function of *d*_obs_ under the condition of *x* in the reservoir model is

$$p\left(d_{\text{obs}}|x\right)\propto \text{exp}\left[-\frac{1}{2}{\left(d_{\text{obs}}-g(x)\right)}^{T}C_{D}^{-1}\left(d_{\text{obs}}-g(x)\right)\right]$$ (5)

Gradient-based optimization and regularization techniques are mainly used toward this goal, generally by maximizing the logarithm of the likelihood and thus, resulting in a single estimated value for a local maximum. A Bayesian approach is adopted herein, which leads not only to a point estimate of the parameters of interest but also to a probability distribution. By attributing a prior distribution to the model parameters and applying the Bayesian theorem, we obtain their posterior distribution, which enables one to quantify the uncertainty concerning the parameter values.

The Bayesian theorem provides the solution of the inverse problem

$$\begin{array}{l} p\left(x|d_{\text{obs}}\right)\propto p\left(d_{\text{obs}}|x\right)p\left(x\right)\\ \propto \text{exp}\left[-\frac{1}{2}{\left(d_{\text{obs}}-g(x)\right)}^{T}C_{D}^{-1}\left(d_{\text{obs}}-g(x)\right)-\frac{1}{2}{\left(x-x_{av}\right)}^{T}C_{M}^{-1}(x-x_{av})\right] \end{array}$$ (6)

where *p*(*x*) is the prior distribution. To obtain the maximum value of the conditional probability of *x* under *d*_obs_, the objective function is

$$P(x)=\frac{1}{2}{\left(x-x_{av}\right)}^{T}C_{X}^{-1}(x-x_{av})+\frac{1}{2}{\left(d_{\text{obs}}-g(x)\right)}^{T}C_{D}^{-1}\left(d_{\text{obs}}-g(x)\right)$$ (7)

When the conditional probability is the maximum value, the corresponding objective function becomes the minimum value. Therefore, reservoir history matching problems can be translated into resolving minimum value problems of the objective function *P*(*x*). When the objective function is the minimum, the corresponding variables *x* are close to the actual parameters.

### Singular value decomposition

For actual reservoir history matching problems, the dimension *N*_*m*_ of the reservoir parameters that require inversion is typically tens of thousands. Therefore, it is extremely difficult to optimize the objective function, and the computational cost can become tremendously expensive due to the large size of the linear systems, especially the large amount of computation that is required to calculate the inverse matrix $C_{X}^{-1}(x-x_{av})$. Therefore, this paper utilizes the singular value decomposition method. Through this method, $C_{X}^{-1}$ and $C_{X}^{-1}(x-x_{av})$ can be transformed into low-dimensional matrixes, which avoids complex computation.

According to the definition of covariance, the covariance matrix of the initial model can be approximately calculated as

$$C_{X}=\frac{1}{N_{e}-1}\sum_{i=1}^{N_{e}}(x_{i}-x_{av}){\left(x_{i}-x_{av}\right)}^{T}=\frac{1}{N_{e}-1}\delta X\delta X^{T}$$ (8)

where $\delta X={\left[\delta x_{1},\delta x_{2},\ldots ,\delta x_{i},\ldots \delta x_{N_{e}}\right]}_{N_{m}\times N_{e}}$, and the *j*th column vector is (*x − x*_*av*_). With Singular Value Decomposition,

$$\delta X=U\Lambda V^{T}$$ (9)

Where *U* and *V* are the singular vectors of *δX*, and Λ is the singular value of *δX*. *U* is composed of orthogonal unit characteristic vectors of *C*_*X*_. Λ^*T*^Λ is composed of characteristic values.

Because $V^{T}V=I_{N_{e}}$,

$$C_{X}^{}\approx \frac{1}{N_{e}-1}U\Lambda \Lambda^{T}U^{T}$$ (10)

Assuming that there are *N*_*S*_ non-zero singular values in Λ, then

$$\Lambda ={\left[\begin{array}{cccc} \sigma_{1}^{} & & & \\ & \sigma_{2} & & \\ & & \ldots & \\ & & & \sigma_{\text{min}(N_{m},N_{e})} \end{array}\right]}_{N_{m}\times N_{e}}={\left[\begin{array}{ccccccc} \sigma_{1} & & & & & & \\ & \sigma_{2} & & & & & \\ & & \ldots & & & & \\ & & & \sigma_{Ns} & & & \\ & & & & 0 & & \\ & & & & & \ldots & \\ & & & & & & 0 \end{array}\right]}_{N_{m}\times N_{e}}=\left[\begin{array}{cc} \Delta & 0\\ 0 & 0 \end{array}\right]$$ (11)

Usually, *N*_*m*_ is far larger than *N*_*e*_. Therefore, *N*_*s*_ is less than or equal to *N*_*e*_. If we only consider the singular value vectors of those models, then

$$C_{X}^{}\approx \frac{1}{N_{e}-1}U_{s}\Lambda_{s}^{2}U_{s}{}^{T}$$ (12)

The approximate inverse matrix of *C*_*X*_ is

$$C_{X}^{-1}=\text{(}N_{e}-1\text{)}U_{s}\Lambda_{s}^{\text{-}2}U_{s}^{T}$$ (13)

By substituting *C*_*X*_ of the objective function with $C_{X}^{-1}$, the *N*_*m*_ dimensional optimization problem about *x* is reduced to *N*_*s*_ dimensions, which avoids the process of solving the inverse of *C*_*X*_ (its dimension can reach up to tens of millions). This method has greatly simplified the difficulty of calculation and improved the computational efficiency. The final function becomes

$$P(x)=\frac{1}{2}{\left(x-x_{av}\right)}^{T}\text{(}N_{e}-1\text{)}U_{s}\Lambda_{s}^{\text{-}2}U_{s}^{T}(x-x_{av})+\frac{1}{2}{\left(d_{\text{obs}}-g(x)\right)}^{T}C_{D}^{-1}\left(d_{\text{obs}}-g(x)\right)$$ (14)

## Solution

### Linear sequential solutions

This paper uses the linear search method for optimization. Here, the linear search consists of two key elements: one is the search step size, which ensures the convergence of the search, and the other is the search direction, which determines the rate of convergence. In actual application, the normalization method of the search direction is usually used for iterative calculation. Now, we introduce the optimization method of the linear search.

In the process of optimizing the objective function *P*(*x*), set the initial variable vector *x* ^0^ to zero and calculate the initial objective function value *P*(*x* ^0^) first. At the *k*th(*k* = 1,2,3,···,*K*_max_) iteration step, do the following: First, set the initial search step *λ* ^*k*^ = 1, and calculate the stochastic gradient ĝ_*k*_(*x*) as the search direction *D* ^*k*^ through the optimization algorithm; then, normalize *D* ^*k*^;

Second, update the variables vector *x* ^*k*^ by $x^{k}=x^{k-1}+\lambda_{k}\frac{D^{k}}{{\left\|D^{k}\right\|}_{\infty }}$;

Third, calculate the objective function value *P*(*x* ^*k*^) and compare *P*(*x* ^*k*^) with *P*(*x* ^k-1^). Then, determine whether *P*(*x* ^*k*^) satisfies $0\leq \frac{\left|P\left(x^{k}\right)-P\left(x^{k-1}\right)\right|}{\text{max}\left\{P\left(x^{k}\right),1\right\}}\leq \varepsilon_{p}$. If the result is YES, then exit the iteration; if the result is NO, then keep the values of *x* ^*k*^ and *P*(*x* ^*k*^), and then, set *k* = *k* + 1 and continue the next iteration step. If *P*(*x* ^*k*^) > *P*(*x* ^k-1^), then reduce *λ*_*k*_ by half and continue to calculate the objective function value *P*(*x* ^*k*^) until it reaches the maximum number of times *m* that *λ*_*k*_ can be halved; if beyond, exit and continue another iteration.

Algorithm I. Linear optimization algorithm.

1. Generate the initial value *x* ^0^.

2. For *k* = 1,2,3,··· until convergence

 (a) Calculate the stochastic gradient ĝ_*k*_(*x*) as the search direction *D* ^*k*^ through the optimization algorithm and set the initial search step to *λ* ^*k*^ = 1.

 (b) For *i* = 1,2,···,*m*, calculate

$x^{k}=x^{k-1}+\lambda_{k}\frac{D^{k}}{{\left\|D^{k}\right\|}_{\infty }}$

 Calculate the objective function value *P*(*x* ^*k*^),

 If *P*(*x* ^*k*^) > *P*(*x* ^k-1^), then check if

$0\leq \frac{\left|P\left(x^{k}\right)-P\left(x^{k-1}\right)\right|}{\text{max}\left\{P\left(x^{k}\right),1\right\}}\leq \varepsilon_{p}$

 If the result is Yes, then exit the iteration.

 If the result is No, then

$\lambda_{k}\text{ = }\frac{\lambda_{k}}{2}$

 End for

End for

In the above algorithm, *ε*_*p*_ is approximately 10^−4^, and ∥•∥_∞_ is the infinite norm value.

Because the stochastic gradient ĝ_*k*_(*x*) in the optimization process is always calculated by the stochastic algorithm, we introduce the stochastic algorithm first.

### Stochastic algorithm based on the directional derivative

The basic principle of this algorithm is to disturb the argument $x={\left[x_{1},x_{2},\cdots ,x_{N_{e}}\right]}^{T}$ and obtain a new variable vector $x\prime ={\left[x_{1}\prime ,x_{2}\prime ,\cdots ,x_{N_{e}}\prime \right]}^{T}$, which is

$$\left[\begin{array}{c} x_{1}\prime \\ x_{2}\prime \\ \vdots \\ x_{N_{e}}\prime \end{array}\right]=\left[\begin{array}{c} x_{1}+\alpha \Delta x_{1}\\ x_{2}+\alpha \Delta x_{2}\\ \vdots \\ x_{N_{e}}+\alpha \Delta x_{N_{e}} \end{array}\right]$$ (15)

Where *α* is the disturbance step, and $\Delta x={\left[\Delta x_{1},\Delta x_{2},\cdots ,\Delta x_{N_{e}}\right]}^{T}$ is the disturbance variables vector, with the element Δ*x*_*i*_(*i* = 1,2,···,*N*_*e*_) in accordance with the Bernoulli distribution of 1 or -1; therefore, $\Delta x_{i}^{-1}$ is equal to Δ*x*_*i*_. By calculating the corresponding objective function values and taking the difference of each increment, we can obtain the stochastic gradient ĝ(*x*).

In each iteration step, ĝ(*x*) of *P*(*x*) at *x* can be expressed as

$$\hat{g}\left(x\right)=\left[\begin{array}{c} \frac{P\left(x_{1}\prime \right)-P\left(x_{1}\right)}{\alpha \Delta x_{1}}\\ \frac{P\left(x_{2}\prime \right)-P\left(x_{2}\right)}{\alpha \Delta x_{2}}\\ \vdots \\ \frac{P\left(x_{N_{e}}\prime \right)-P\left(x_{N_{e}}\right)}{\alpha \Delta x_{N_{e}}} \end{array}\right]$$ (16)

The stochastic gradient is

$$\hat{g}\left(x\right)=\frac{P\left(x+\alpha \Delta x\right)-P\left(x\right)}{\alpha }\times \Delta x^{\text{-}1}$$ (17)

The directional derivative reflects the rate of change of the objective function value in a specific direction. The formulation is

$$\begin{array}{l} \frac{\partial P}{\partial l}=\frac{P(x+\alpha \Delta x)-P\left(x\right)}{\sqrt{{\left(\alpha \Delta x_{1}\right)}^{2}+{\left(\alpha \Delta x_{2}\right)}^{2}+\cdots +{\left(\alpha \Delta x_{N_{e}}\right)}^{2}}}=\frac{P(x+\alpha \Delta x)-P(x)}{\alpha \Delta x}\times \frac{\Delta x}{{\left\|\Delta x\right\|}_{2}}\\ \;={\hat{g}}_{1}\cdot \text{cos}\varphi_{1}+{\hat{g}}_{2}\cdot \text{cos}\varphi_{2}+\cdots {\hat{g}}_{N_{e}}\cdot \text{cos}\varphi_{N_{e}}={\left(\text{cos}\langle \varphi \rangle \right)}^{T}\cdot \hat{g} \end{array}$$ (18)

where $\frac{\partial P}{\partial l}$ is the directional derivative of the objective function in the $\vec{l}$ direction; and cos*θ*_*i*_ is the cosine value of $\langle e_{i},\vec{l}\rangle$.

The directional derivative of *x* by using the true gradient can be expressed as

$$\frac{\partial P}{\partial l}={\hat{g}}_{1}\cdot \text{cos}\varphi_{1}+{\hat{g}}_{2}\cdot \text{cos}\varphi_{2}+\cdots {\hat{g}}_{N_{e}}\cdot \text{cos}\varphi_{N_{e}}={\left(\hat{g}\right)}^{T}\text{cos}\langle \varphi \rangle$$ (19)

From Eqs 18 and 19, we can obtain

$${\left(\frac{\partial P}{\partial l}\right)}^{2}={\left(\hat{g}\right)}^{T}\cdot \text{cos}\langle \varphi \rangle \cdot {\left(\text{cos}\langle \varphi \rangle \right)}^{T}\cdot \hat{g}\geq 0$$ (20)

Set $g\prime =\text{cos}\langle \varphi \rangle \cdot {\left(\text{cos}\langle \varphi \rangle \right)}^{T}\cdot \hat{g}$. From Eq 20, we can see that *g*’ is the increasing direction, which ensures the convergence of this algorithm.

We usually obtain the average of the stochastic gradient through several disturbances in pursuit of improving the accuracy of the gradient, and then, we resolve the problem by the above optimization method (Algorithm I) after obtaining a stochastic gradient.

$$\bar{g}\left(x\right)=\frac{\sum_{k=1}^{N}g_{k}\prime }{N}$$ (21)

Algorithm II. Stochastic algorithm based on directional derivative.

1. For *k* = 1,2,···*N*, generate random Δ*x* ^*k*^

 (a) Calculate *x* ^*k*^ = *x* + *α*Δ*x* ^*k*^ and *p*(*x* ^*k*^),

 (b) Then, compute

${\hat{g}}_{k}=\frac{P\left(x^{k}\right)-P\left(x\right)}{\alpha \Delta x^{k}}$

 (c) Calculate the cosine value cos*φ*.

 (d) Set $g_{k}\prime =\text{cos}\langle \varphi \rangle \cdot {\left(\text{cos}\langle \varphi \rangle \right)}^{T}\cdot {\hat{g}}_{k}$.

 End for

(2) Calculate $\bar{g}=\sum_{k=1}^{N}g_{k}\prime /N$

Because the gradient obtained from Algorithm II is approximate, the stochastic gradient with a perturbation and directional derivative has a high uncertainty. Although Algorithm II has improved some of the shortcomings that other algorithms have, it also has many deficiencies, such as a large number of iterations and slow convergence of the objective function. To avoid these shortcomings, we propose a new algorithm by modifying Algorithm II.

### Principle of the hybrid algorithm

The main idea of the Hybrid algorithm is to first calculate the stochastic gradient of the objective function by using Algorithm II and, then, to replace the components with the largest (in magnitude) stochastic gradient with approximate values of the gradient from the finite difference in a proper sequence until the direction of the modified gradient is nearly the same as the unknown real gradient direction.

According to Taylor’s formula, *P*(*x*+*α*Δ*x*) − *P*(*x*) can be expanded as

$$\begin{array}{ll} P\left(x+\alpha \Delta x\right)-P\left(x\right) & \approx \alpha \Delta x\frac{\partial P(x)}{\partial x}\\ & =\alpha \overset{N_{e}}{\underset{k=1}{\sum }}\Delta x_{k}\frac{\partial P(x)}{\partial x_{k}}\\ & =\alpha {\left(\Delta x\right)}^{T}\nabla P(x) \end{array}$$ (22)

Then,

$$\begin{array}{ll} \frac{\partial P(x)}{\partial x_{k}} & =\frac{P\left(x+\alpha \Delta x_{k}e_{k}\right)-P\left(x\right)}{\alpha \Delta x_{k}e_{k}}\\ & \approx \frac{P\left(x+\alpha \Delta x_{k}e_{k}\right)-P\left(x\right)}{\alpha \Delta x_{k}} \end{array}$$ (23)

where *e*_*k*_ is a unit vector in the *k*th direction. Substituting Eq 23 into Eq 22, we obtain

$$\begin{array}{ll} \Delta P\left(x\right) & =P\left(x+\alpha \Delta x\right)-P\left(x\right)\\ & \approx \overset{N_{e}}{\underset{k=1}{\sum }}\left[P\left(x+\alpha \Delta x_{k}e_{k}\right)-P\left(x\right)\right]\\ & =\overset{N_{e}}{\underset{k=1}{\sum }}\Delta P_{k}(x) \end{array}$$ (24)

Here, Δ*P*_*k*_(*x*) is the increment of the objective function value in the *k*th direction.

The conclusion is that the increment of the objective function value with the simultaneous perturbation of all of the components is approximately equal to the sum of each function value increment with the separate disturbance of each component of the vector *x*.

### Hybrid algorithm

A simple equation is introduced to vertify the changement og some components has a great impact on the result of function.

$$y(x)=(x-a){\left(x-a\right)}^{T}$$ (25)

Where *a* = [1,1,1,1,1]. If *x*_0_ = [2,3,2.5,5,1], then we will obtain *y*(*x*_0_) = 23.25. Set Δ*x* = [0.5,0.5,0.5,0.5,0.5] and calculate the function value of *x*_0_ + Δ*x* and the increments of the function value with each component.

From Table 1, we can realize that Δ*y*(*x*) takes the maximum value when the 4th component is changed, which has the greatest impact on the result. Here, $x_{0}^{\left(4\right)}$ is the largest component of all of the components, and thus, we regard $x_{0}^{\left(4\right)}$ as the ‘important’ element. We could find out the ‘relatively important’ elements of the stochastic gradient by relying on this standard.

**Table 1:** The increment of the function value at each component of *x*

Δ*y*(*x*_1_)	$\Delta y(x_{1}^{\left(1\right)})$	$\Delta y(x_{1}^{\left(2\right)})$	$\Delta y(x_{1}^{\left(3\right)})$	$\Delta y(x_{1}^{\left(4\right)})$	$\Delta y(x_{1}^{\left(5\right)})$
9.75	1.25	2.25	1.75	4.25	0.25

Here, $x_{1}^{\left(i\right)}$ is the increment of the *i*th(*i* = 1,2,3,4,5) component of *x*_0_.

The index collection of all of the components in the stochastic gradient can be denoted as *U* = {1,2,···,*N*_*e*_}; *I*_*u*_ and *I*_*d*_ are its two subsets, which satisfy *I*_*u*_ ∪ *I*_*d*_ = *U* and *I*_*u*_ ∩ *I*_*d*_ = *ϕ*.*I*_*u*_ denotes the relatively ‘important’ components of the stochastic gradient, and *I*_*d*_ denotes the ‘unimportant’ components.

The hybrid algorithm is used to calculate the elements of *I*_*u*_ with the finite difference method and replace the elements of *I*_*d*_ by the stochastic gradient.

Determining the search direction is a process of continuous iteration; thus, we set the initial values $I_{u}^{0}=\phi$ and $I_{d}^{0}=U$, $N_{u}^{0}=0$ and $N_{d}^{0}=N_{e}$. $N_{u}^{0}$ and $N_{d}^{0}$ denote the number of elements in $I_{u}^{0}$ and $I_{d}^{0}$, respectively, and the superscript denotes the iteration step.

From Eq 22, we can obtain

$$\Delta P_{u}^{\left(j\right)}(x)=\alpha \sum_{k\in I_{u}}\Delta x_{k}\frac{\partial P(x)}{\partial x_{k}}$$ (26)

$$\Delta P_{d}^{\left(j\right)}(x)=\alpha \sum_{k\in I_{d}}\Delta x_{k}\frac{\partial P(x)}{\partial x_{k}}$$ (27)

where *j* is the number of samples Δ*P* ^j^(*x*), *j* = 1,2,···*N*_max_.

When *m* = 0, $\Delta P_{u}^{(j),0}(x)$ is equal to zero, and $\Delta P_{d}^{(j),0}(x)=\Delta P^{\left(j\right)}(x)$. At the *m*th iteration step,

$$\Delta P^{\left(j\right)}(x)=\Delta P_{u}^{(j),m}(x)+\Delta P_{d}^{(j),m}(x)$$ (28)

Now, we consider the *m*th iteration step.

**First**, generate a stochastic gradient by using Algorithm II:

$$\begin{array}{ll} \hat{g}(x^{k}) & =\frac{1}{N_{\text{max}}}\overset{N_{\text{max}}}{\underset{j=1}{\sum }}\left[\frac{P(x^{k}+\alpha e^{\left(j\right)})-P(x^{k})}{\alpha }\right]e^{\left(j\right)}\text{cos}\left(\varphi \right)\\ & =\frac{1}{N_{\text{max}}}\overset{N_{\text{max}}}{\underset{j=1}{\sum }}\left[\frac{\Delta P^{\left(j\right)}(x^{k})}{\alpha }\right]e^{\left(j\right)}\text{cos}\left(\varphi \right) \end{array}$$ (29)

The *l*th component of the gradient ĝ(*x* ^*k*^) is

$${\hat{g}}_{l}(x^{k})=\frac{1}{N_{\text{max}}}\sum_{j=1}^{N_{\text{max}}}\frac{\Delta P^{\left(j\right)}(x^{k})}{\alpha }e_{l}^{\left(j\right)}\text{cos}\left(\varphi \right)$$ (30)

Replace Δ*P* ^(j)^ with Δ*P* ^(j),m^; then,

$${\hat{g}}_{l}(x^{k})=\frac{1}{N_{\text{max}}}\sum_{j=1}^{N_{\text{max}}}\frac{\Delta P^{(j),m}(x^{k})}{\alpha }e_{l}^{\left(j\right)}cos\left(\varphi \right),l\in I_{d}^{m}$$ (31)

Therefore, we can use Eq 13 to calculate the *l*th component of the stochastic gradient in $I_{d}^{m}$.

**Second**, calculate each component of the stochastic gradient ĝ(*x* ^*k*^) and sort them according to their absolute values. Take the first *N*_*key*_ elements as the ‘important’ elements, and record their subscripts in $I_{u}^{m+1}$; then, record the subscripts of the remaining elements in $I_{d}^{m+1}$, and update $I_{u}^{m+1}$ and $I_{d}^{m+1}$.

**Third**, recalculate the *i*th(*i* = 1,2,···,*N*_*key*_) ‘important’ element in $I_{u}^{m+1}$ by the finite difference approach:

$$\frac{\partial P(x^{k})}{\partial x_{i}}=\frac{P(x^{k}+\alpha e_{i})-P(x^{k})}{\alpha },i\in I_{u}^{m+1}$$ (32)

Then, obtain the realistic gradient $\nabla P_{u}^{m+1}(x^{k})$, where the *i*th element ($i\in I_{u}^{m+1}$) can be obtained by finite differences, and record the other elements as zero.

Calculate $\Delta P_{u}^{(j),m+1}$ and $\Delta P_{d}^{(j),m+1}$

$$\Delta P_{u}^{(j),m+1}(x^{k})=\sum_{i\in I_{u}^{\left(j\right)}}\alpha e_{l}^{\left(j\right)}\frac{\partial P(x^{k})}{\partial (x_{i})}$$ (33)

$$\Delta P_{d}^{(j),m+1}(x^{k})=\Delta P^{(j),m+1}(x^{k})-\Delta P_{u}^{(j),m+1}(x^{k})$$ (34)

Where *j* = 1,2,···*N*_max_.

**Fourth**, calculate the angle *θ* between the approximate gradient and the unknown true gradient, as follows:

$$\text{cos}\left(\theta \right)=\sqrt{1-\frac{E\left[{\left(\Delta P_{d}^{m}(x^{k})\right)}^{2}\right]}{E\left[{\left(\Delta P(x^{k})\right)}^{2}\right]}}$$ (35)

If cos(*θ*) ≥ *ε*, then set $D^{k}=\nabla P_{u}^{m}$ and exit the Loop.

If cos(*θ*) < *ε*, then determine whether the ‘important’ elements in $I_{u}^{m}$ obtained by the stochastic gradient correspond to the important elements of the true gradient. Check each *i*th element in the collection $I_{u}^{m}$, as follows:

$${\left(\frac{P(x^{k}+\alpha e_{i})-P(x^{k})}{\alpha }\right)}^{2}\geq \frac{1}{N_{\text{max}}}\frac{E[{\left(\Delta P(x^{k})\right)}^{2}]}{\alpha^{2}}$$ (36)

If there exists one component of the stochastic gradient that is ‘important’, but the value recalculated by finite differences does not satisfy Eq 36, then mark this identification of the ‘important’ component as *failure* and add the number of *failure* times to 1. The maximum number of *failure* times allowed in the *m*th iteration step is *N*_*f*,max_.

If the number of *failure* times *N*_*i*_ exceeds *N*_*f*,max_, then we should reselect new samples, recalculate the significant elements and determine the ‘important’ elements. If *N*_*i*_ < *N*_*f*,max_, then reselect *N*_*key*_‘important’ elements from $I_{d}^{m}$ by finite differences, and set *m* = *m*+1; continue this iteration until cos(*θ*) ≥ *ε*.

### The standard of judgment

As mentioned earlier, *P*_*u*_ is the approximation gradient that is in almost the same direction as the true gradient. The cosine of the angle between these two gradients is given by

$$\text{cos}\left(\theta \right)=\frac{{\left(\nabla P_{u}\right)}^{T}\nabla P}{\left\|\nabla P_{u}\right\|\cdot \left\|\nabla P\right\|}$$ (37)

where ∇*P*_*u*_ is the approximation gradient that is obtained by using finite differences, and ∇*P* is the unknown true gradient.

Set ∇*P*_*u*,*l*_(*x*) to be the *l*th component of ∇*P*(*x*). When the element *l* satisfies *l* ∈ *l*_*u*_ in ∇*P*(*x*), then calculate ∇*P*(*x*) by finite differences; if *l* ∈ *l*_*d*_, then set it to zero.

$$\nabla P_{u,l}\left(x\right)=\left\{ \begin{array}{r} \frac{P\left(x^{k}+\alpha e_{l}\right)-P\left(x^{k}\right)}{\alpha },l\in I_{u}\\ 0,l\in I_{d} \end{array}\right.$$ (38)

Then,

$$\begin{array}{ll} {\left(\nabla P_{u}\right)}^{T}\nabla P & =\sum_{}\nabla P_{u,l}\frac{\partial P(x^{k})}{\partial x_{l}}\\ & \approx \sum_{}{\left[\frac{P(x^{k}+\alpha e_{l})-P(x^{k})}{\alpha }\right]}^{2}\\ & \approx \sum_{}{\left[\nabla P_{u,l}(x^{k})\right]}^{2}\\ & \approx \|\nabla P_{u}{\|}^{2} \end{array}$$ (39)

Replacing (∇*P*_*u*_)^T^ ∇*P* by ∥∇*P*_*u*_∥^2^ in Eq 37,

$$\text{cos}\left(\theta \right)\approx \frac{{\left\|\nabla P_{u}\right\|}^{2}}{\left\|\nabla P_{u}\right\|\left\|\nabla P\right\|}=\frac{\left\|\nabla P_{u}\left(x^{k}\right)\right\|}{\left\|\nabla P\left(x^{k}\right)\right\|}$$ (40)

with

$$\Delta P(x^{k})=\alpha Z^{T}\nabla P(x^{k})=\alpha \nabla P{\left(x^{k}\right)}^{T}Z$$ (41)

Because *Z* obeys the Gaussian distribution, therefore

$$E(ZZ^{T})=I$$ (42)

Then,

$$\begin{array}{ll} E[{\left(\Delta P\left(x^{k}\right)\right)}^{2}] & =\alpha^{2}\nabla P{\left(x^{k}\right)}^{T}\nabla P\left(x^{k}\right)\\ & =\alpha^{2}\|\nabla P\left(x^{k}\right){\|}^{2} \end{array}$$ (43)

with

$${\left\|\nabla P(x^{k})\right\|}^{2}={\left\|\nabla P_{u}(x^{k})\right\|}^{2}+{\left\|\nabla P_{d}(x^{k})\right\|}^{2}$$ (44)

and

$$E[{\left(\Delta P_{d}\right)}^{2}]=\alpha^{2}{\left\|\nabla P_{d}(x^{k})\right\|}^{2}$$ (45)

We can obtain

$$\begin{array}{ll} \text{cos}\left(\theta \right) & =\sqrt{\frac{\|\nabla P_{u}\left(x^{k}\right){\|}^{2}}{\|\nabla P\left(x^{k}\right){\|}^{2}}}\\ & =\sqrt{\frac{E[{\left(\Delta P(x^{k})\right)}^{2}]-E[{\left(\Delta P_{d}(x^{k})\right)}^{2}]}{E[{\left(\Delta P(x^{k})\right)}^{2}]}}\\ & =\sqrt{1-\frac{E[{\left(\Delta P_{d}(x^{k})\right)}^{2}]}{E[{\left(\Delta P(x^{k})\right)}^{2}]}} \end{array}$$ (46)

The angle between the approximation gradient and the true gradient can be calculated by Eq 46.

According to Pradlwarter (2007)[27], to determine whether the *l*th component of the gradient in *I*_*u*_ is ‘important’, we can calculate the square value of this component and compare it with the average of all of the components of ∇*P*(*x* ^*k*^), as follows:

$${\left(\frac{\partial P(x^{k})}{\partial x_{l}^{k}}\right)}^{2}\geq \frac{{\left\|\nabla P(x^{k})\right\|}^{2}}{N_{\text{max}}}$$ (47)

If the result is ‘YES’, then mark it as ‘important’. Conversely, if the result is ‘NO’, then consider it to be unimportant. Because of the unknown true gradient ∇*P*(*x* ^*k*^), according to Eq 43, Eq 47 can be expressed as

$${\left(\frac{P\left(x^{k}+\alpha e_{l}\right)-P\left(x^{k}\right)}{\alpha }\right)}^{2}\geq \frac{1}{N_{\text{max}}}\frac{E\left[{\left(\Delta P\left(x^{k}\right)\right)}^{2}\right]}{\alpha^{2}}$$ (48)

With the above equation, we can determine whether the *l*th element is ‘important’ or not.

### The verification and improvement

#### Function I

The first mathematical function is to illustrate the reliability of the Hybrid algorithm. The objective is to minimize the following function:

$$P\left(x\right)=\sum_{i=1}^{N}10x_{i}^{2}$$ (49)

Where *x* = [*x*_1_,*x*_2_,···*x*_*N*_]^*T*^, and the initial guess is *x*_*i*_ = 2,*i* = 1,···,*N*. We set the number of variables to be *N* = 100. The minimum of Eq 49 is 0, which occurs when *x* = [0,0,···0]^*T*^.

In Algorithm II, set the initial search step to *λ*_0_ = 1, regard the average of the stochastic gradient calculated with 5 stochastic perturbations as the search direction, and set the disturbance variable *α* to 0.015. Record the objective function values of each iteration step and the cosine values.

In the Hybrid algorithm: (1) Obtain the stochastic gradient by using Algorithm II, and then, select 5 ‘important’ components of the stochastic gradient, which constitutes a new approximate gradient to iterate; (2) The angle between the approximate gradient and the true gradient should satisfy cos(*θ*) ≥ 0.8. If not, then recalculate and reselect the ‘important’ elements; (3) The maximum number of *failure* allowed is 2; (4) The maximum number of simulations is 25; if this number is exceeded, then recalculate the stochastic gradient. Calculate the objective function value for the optimization in both Algorithm II and the Hybrid algorithm, and compare the rate of convergence and cos(*θ*) for the two methods.

Table 2 shows the behavior and results obtained from Hybrid algorithm for the first iteration *m* = 1. The first column refers to the iteration index *s* during the calculation of the approximate gradient ∇*P*_*u*_, and two iterations are used to obtain ∇*P*_*u*_ until satisfies cos(*θ*) ≥ 0.8. Column 2 to Column 5 show that five components of the gradient from Algorithm II are recomputed by using finite difference at each iteration, meanwhile, the component index and the specific gradient values are shown in column 2, column3 and column 4, respectively. Because the gradient is particular stochastic, the chosen components of the gradient by Algorithm II may not necessarily refer to the actual “important components”. As shown at iteration s = 1, the 71th component value of the gradient by Algorithm II is as large as -1613.82 while the recomputed approximate gradient is a much smaller 81, notice that in Eq 47, the 71th component turns out to be *failure*. When the *failure* time reaches the maximum allowable number 2, it suggests that the approximate gradient is not accurate enough, then continue another iteration until cos(*θ*) ≥ 0.8. During the two iterations, components 71,128,55,183,85,10,25,116,70 and 45 are recomputed by finite difference and the rest components of are set equal to zero.

**Table 2:** Behavior of the Hybrid algorithm.

Iteration Index s	Component Index *i*	Algorithmm II	Hybrid algorithm	Flag	Failure time	cos(*θ*)
1	71	-1613.82	81	Failure		
	128	-1496.35	65	Failure		
	55	1223.8	821	Success		
	183	-756.242	185	Success		
	85	687.782	1880	Success	2	0.59
2	10	594.311	2200	Success		
	25	734.968	520	Success		
	116	-660.272	81	Failure		
	70	284.032	1040	Success		
	45	703.37	560	Success	1	0.85

**Fig 2:**
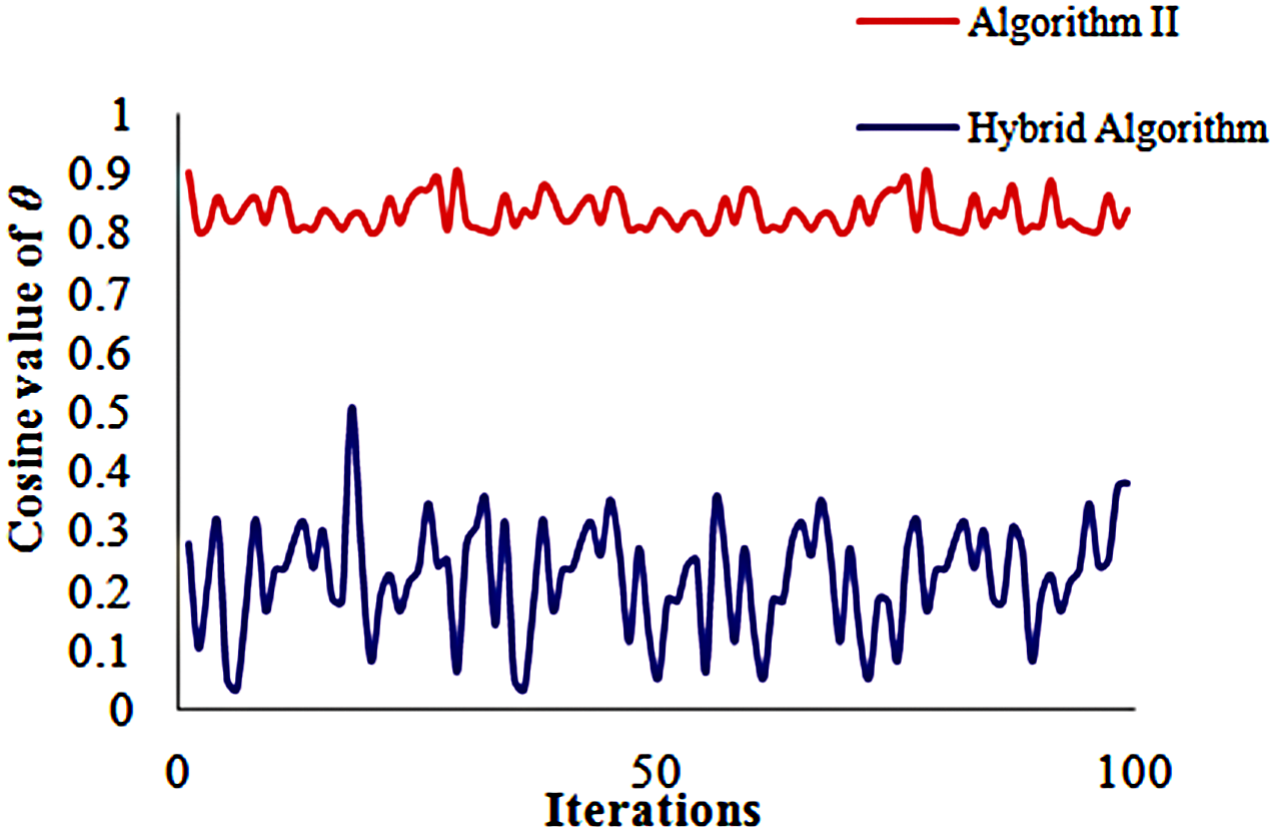
Accuracy of cos(*θ*) of both algorithms. The blue line represents the cosine value calculated by Hybrid algorithm and the red line represents the cosine value calculated by Algorithm II.

Fig 2 shows that the cosine value of *θ* obtained by Algorithm II is approximately 0.3, which illustrates that the angle between the approximate gradient and the true gradient is approximately 73 degrees; however, the cosine value of *θ* obtained by the Hybrid algorithm is larger than 0.8 (in other words, the angle between the approximate gradient and the true gradient does not more than 35 degrees). The result has demonstrated that the direction of the gradient from the Hybrid algorithm is closer to the true gradient direction than it from Algorithm II, which illustrates that the Hybrid algorithm has a higher accuracy than Algorithm IIin the search direction at each iteration step.

**Fig 3:**
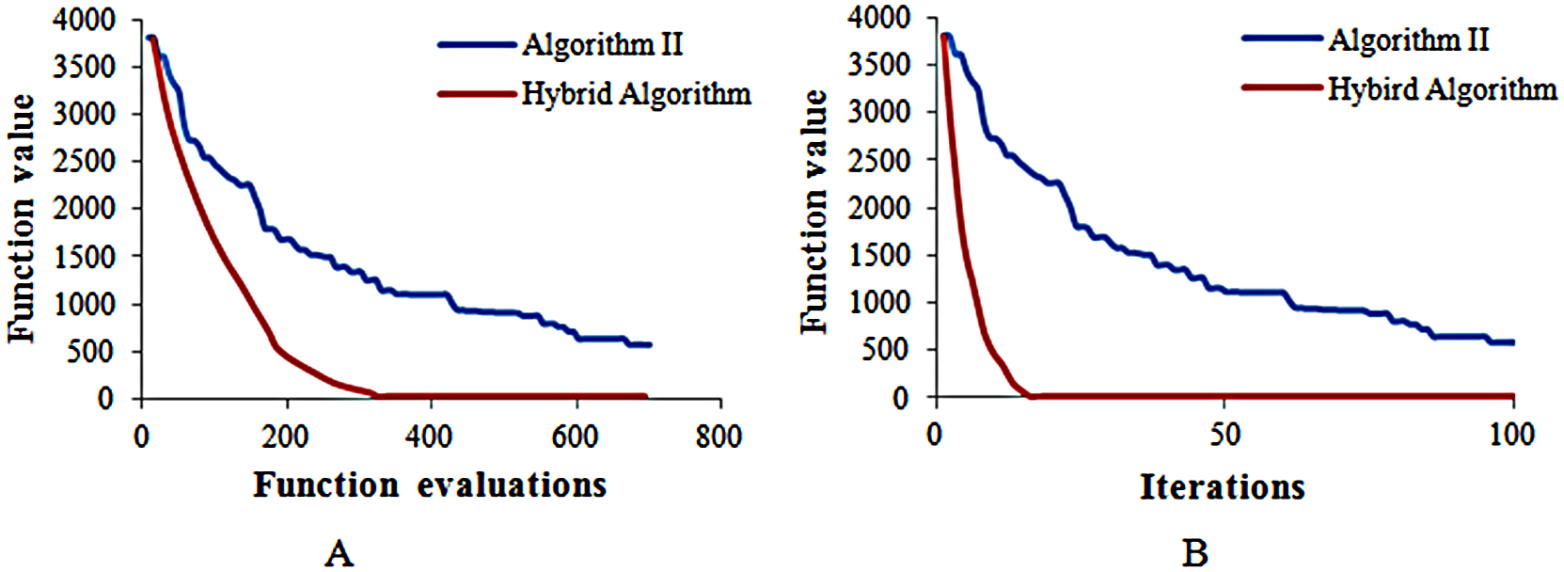
The convergence of the objective function. A:The objective function value versus function evaluations. B: The objective function value versus function iterations.

Fig 3(A) shows that the objective function value will decrease in both Algorithm II and the Hybrid algorithm. This finding means that the objective function also converges with the Hybrid algorithm. From the above Fig, we can find that the Hybrid algorithm behaves better than Algorithm II and has a higher rate of convergence. At approximately the 400th computation time in the Hybrid algorithm, the objective function value is close to the final value, while Algorithm II requires probably over 800 computation times to reach the same value. Thus, the Hybrid algorithm reaches the same result of convergence as Algorithm II with less simulation runs.

Fig 3(B) shows that to achieve the same value of the objective function, Algorithm III requires 20 iteration steps, while Algorithm II requires more than 100 iteration steps.

#### Function II

The Rosenbrock function is a nonconvex function that is often used to test the performance of an optimization algorithm. Hence, the objective is to minimize the Rosenbrock function for the N-variable case, which is given by

$$P\left(x\right)=\sum_{i=1}^{N/2}\left[{\left(x_{2i}-x_{2i-1}^{2}\right)}^{2}\text{+}{\left(1-x_{2i-1}\right)}^{2}\right]$$ (50)

Where *x* = [*x*_1_,*x*_2_,··· *x*_*N*_]^T^, and the initial guess is *x*_*i*_ = 2,*i* = 1,···,*N* We consider four cases, in which the number of variables is *N* = 100,200,400,800, and we compare the optimization results by using the Hybrid algorithm. In each step, we require the accuracy in the direction of the estimated gradient to satisfy cos(*θ*) ≥ 0.8.

**Fig 4:**
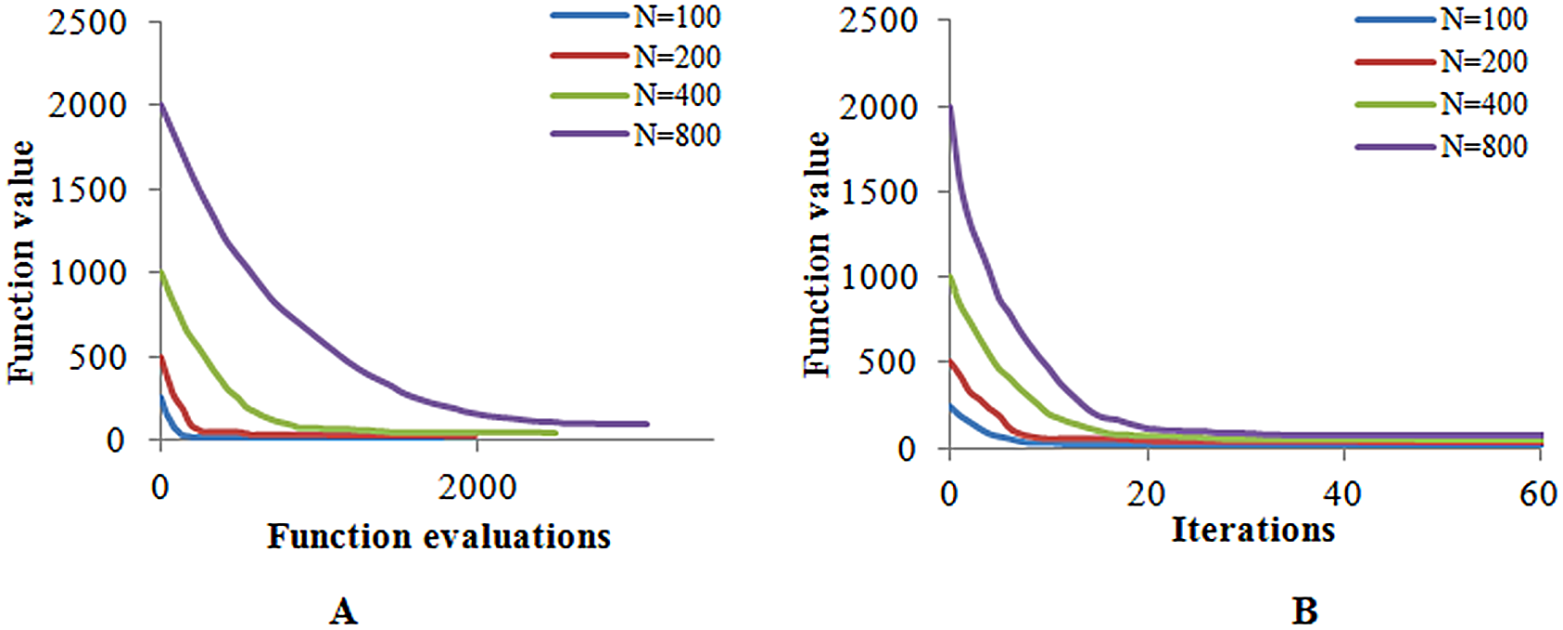
The optimization results in different cases. Each line represents the convergence of the objection function in different numbers of the variables.

Fig 4(A) shows that the objective function can obtain a minimum value with the Hybrid algorithm in different cases, and the function evaluations increase with the increase in the number of variables. We also could attain the same property from Fig 4(B). The result has verified that this algorithm is acceptable with a different number of variables.

Next, we consider the four cases with respect to both Eqs 49 and 50, to optimize and compare the cosine values and finite difference frequency (Table 3). From Table 3, we can realize that the estimated gradient by the Hybrid algorithm satisfies cos(*θ*) ≥ 0.8 with different numbers of variables; the finite difference (F-D) times are not the same in different steps, and the average number of finite differences increases with the increase in the number of variables.

**Table 3:** The contradistinction results in different cases.

Equation	Example Index *i*	The number of variables	The average cosine value	Max time (F-D)	Min time (F-D)	Average time ineach step (F-D)
49	1	100	0.85602	35	5	14
	2	200	0.82892	50	10	27
	3	400	0.81852	105	15	51
	4	800	0.81554	180	25	94
50	5	100	0.83359	45	5	17
	6	200	0.82296	55	10	26
	7	400	0.81859	110	15	49
	8	800	0.81549	220	20	98

From Table 3, we can realize that the finite difference frequency can be excessive, which would reduce the convergence in a certain iteration step, while in another iteration step, the finite difference frequency could be very small. Because of this uncertainty, we introduce the average number of finite differences in each step instead. Now, we discuss the relationship between the differential rate and the number of variables.

**Fig 5:**
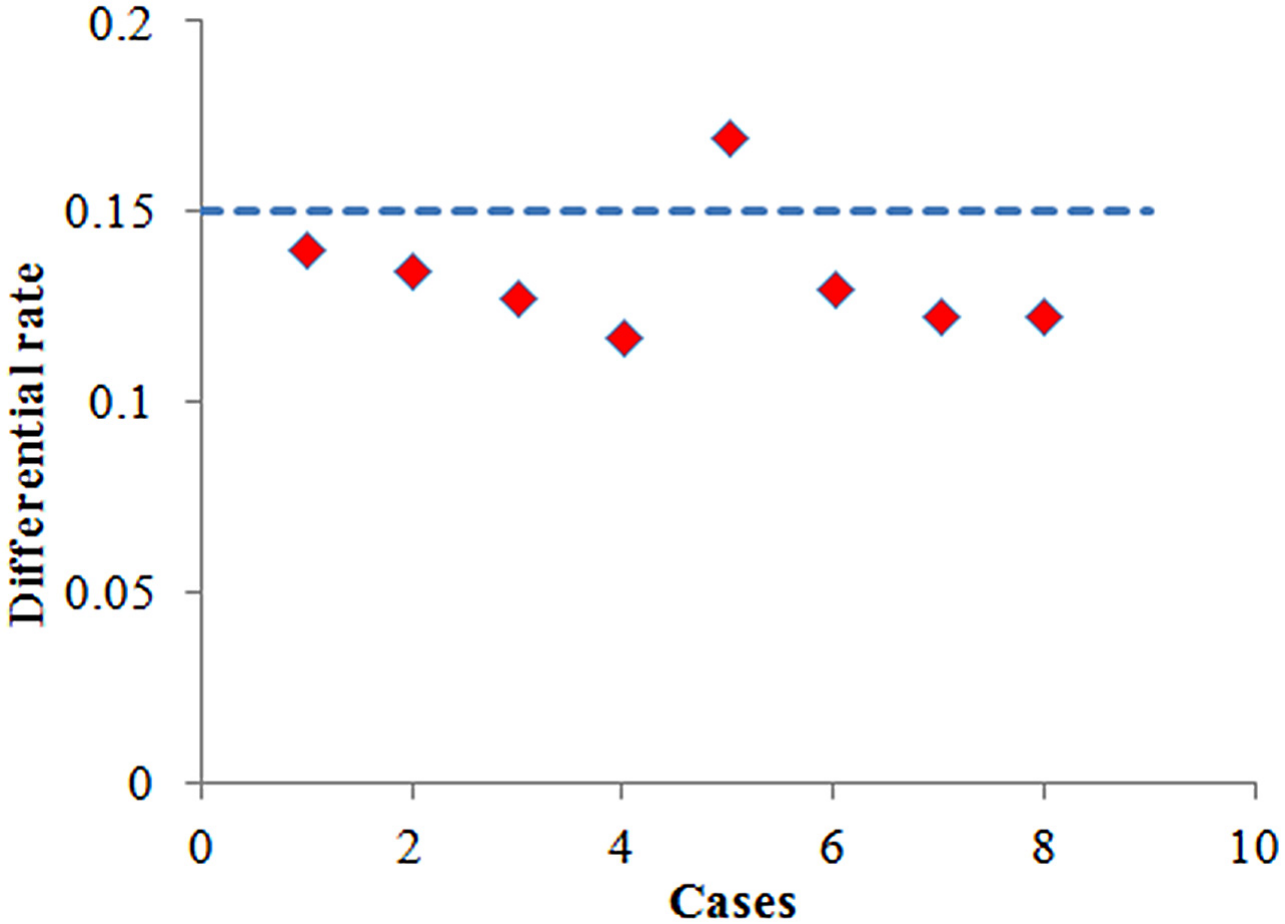
The differential rate in different cases. There are a total of eight kinds of cases. The maximum value is no more than 20% and The minimum value is no less than 10%.

Fig 5 illustrates the differential rate (the ratio of the difference frequency and the number of variables in each step) in different cases, which we have discussed. Through this Fig, we can obtain that in most cases, the differential rate is less than 0.15, which means that we can obtain a much more accurate gradient by finite differences for 15 times per 100 variables. Thus, we take 15% of the number of all the variables for finite differences to replace the judgment of the cosine value.

Because we use quantitative finite differences to replace the judgment of the cosine value in every step, the Hybrid algorithm can become the following:

Algorithm III. The improvement of the Hybrid algorithm.

1. Initialization: *I*_*u*_ = *ϕ* and *I*_*d*_ = *U*,*N*_*u*_ = *0* and N_*d*_ = N_*e*_.

2. For *j* = 1,2,···*N*_max_, generate *N*_max_ samples.

Calculate ĝ(*k*) by using Algorithm II.

3. Compute each component of ĝ(*k*) and sort them, and then, filter the front 0.15*N*_*e*_ elements into *I*_*u*_ and put the remaining elements into *I*_*d*_.

4. Replace the elements of *I*_*u*_ with the elements by finite difference and set the other elements to zero.

5. Generate a new gradient to replace ĝ(*k*).

According to the above analysis, we can realize the following: (1) the approximate gradient obtained by introducing finite differences into Algorithm II has better convergence than that generated only by Algorithm II; (2) With fewer iterations and less computation time, the Hybrid algorithm has a better optimization result than Algorithm II; and (3) the quantitative finite difference frequency can improve the stability of the Hybrid algorithm. Those characteristics provide the possibility for the application of solving the inverse problem of reservoir history matching.

## Numerical Examples

### Case1: Theoretical reservoir model

**Fig 6:**
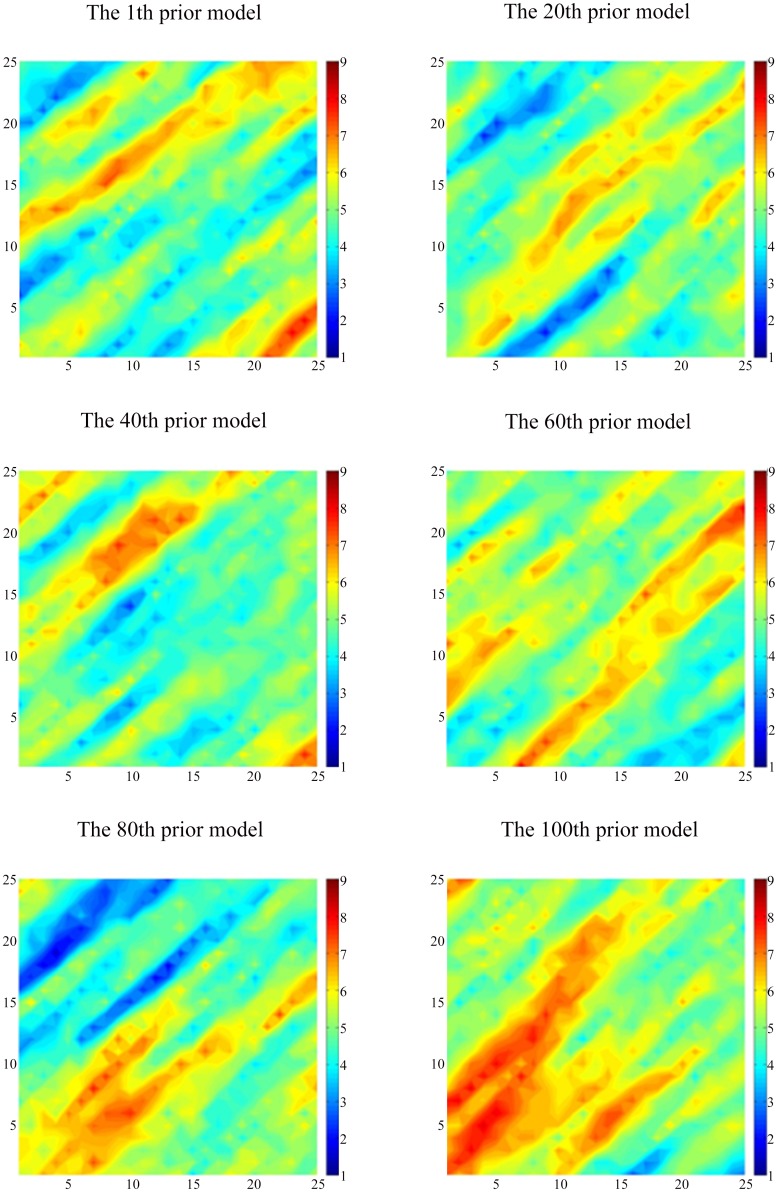
The log-permeability distribution of the prior models. These models are generated by the professional geological modeling software: Petrel.

This reservoir simulation model is a simple 2D model with 25×25 two-dimensional log-permeability distributions with great heterogeneity. It includes a total of 9 injection wells and 4 production wells. In this model, the period of history matching is 1200 days, and every control step is 120 days. In other words, the well control parameter will be adjusted per every 120 days. In the process of history matching, the permeability at each of the 625 cells in the grid must be calculated and updated. The production data required for matching includes the flowing bottom-hole pressure and the oil and water production of the wells. We can regard the simulation result as the observed data of the reservoir by adding the errors of the normal distribution to the result of the initial model. We generate 100 initial reservoir models based on prior geological information by using the Sequential Gaussian Simulation Method (Fig 6).

From Fig 6, we can see that there exist large differences between the distributions of the initial permeability among those models; the hypertonic and hypotonic zones are quite different. However, every model has reflected the basic reservoir characteristics: the direction of the hypertonic stripes and their approximate positions. By generating 100 stochastic models have reduced the construction error of the models because of their differences.

**Fig 7:**
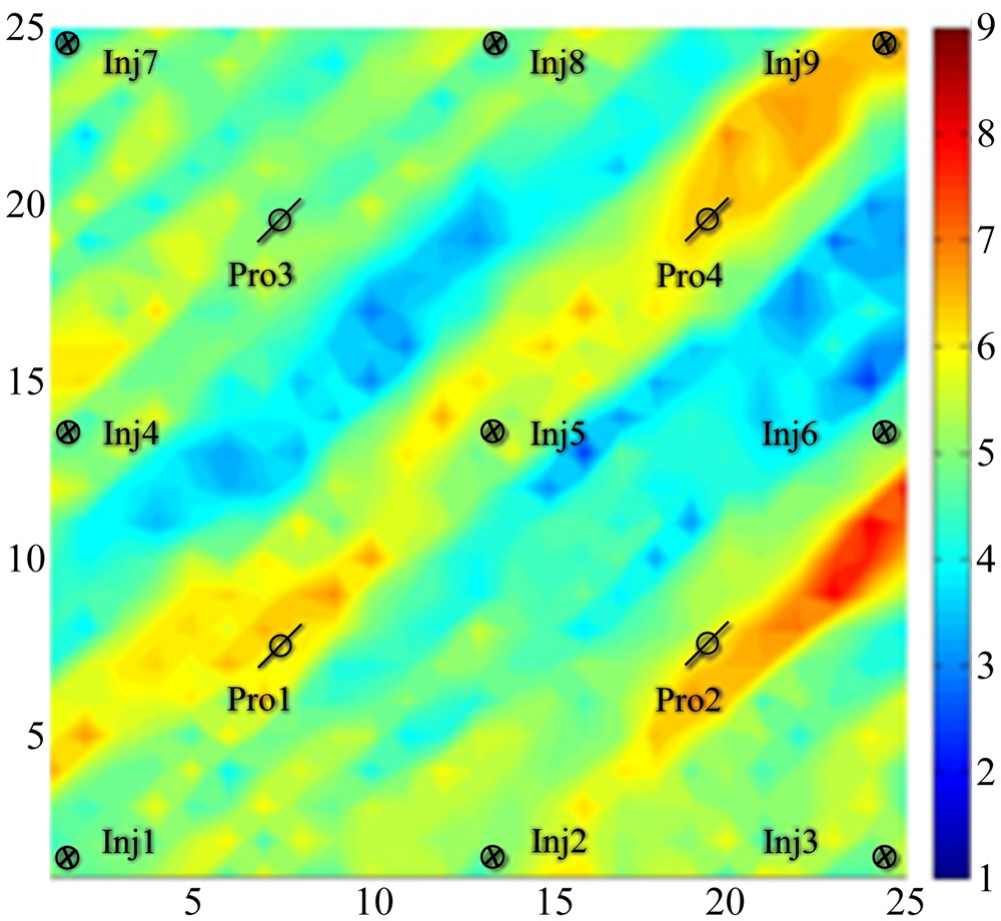
The real log-permeability distribution. This reservoir uses five spot pattern for numerical simulation.

Fig 7 shows the real permeability field which generated from 100 random reservoir models. Because the parameters in those models are in accordance with Gaussian distributions, the real permeability (Fig 7) is also in accordance with Gaussian distributions. We use Algorithm II and III for the optimization of history matching objective function, and then compare the optimization results.

To obtain the minimum value of Eq 14, we use both Algorithm II and Algorithm III. In Algorithm II: We set the initial step to 1 in the linear search process and obtain the stochastic gradients, which requires 5 random perturbations at each step; then, we iterate with their average gradient.

In Algorithm III: The initial setting and the process of generating the stochastic gradient are consistent with Algorithm II. The difference between the two algorithms is that the gradients for the iterations are not the same. In Algorithm II, the calculated stochastic gradient is used directly for optimization, while in Algorithm III, a new gradient must be generated from the previous stochastic gradient with quantitative finite differences.

**Fig 8:**
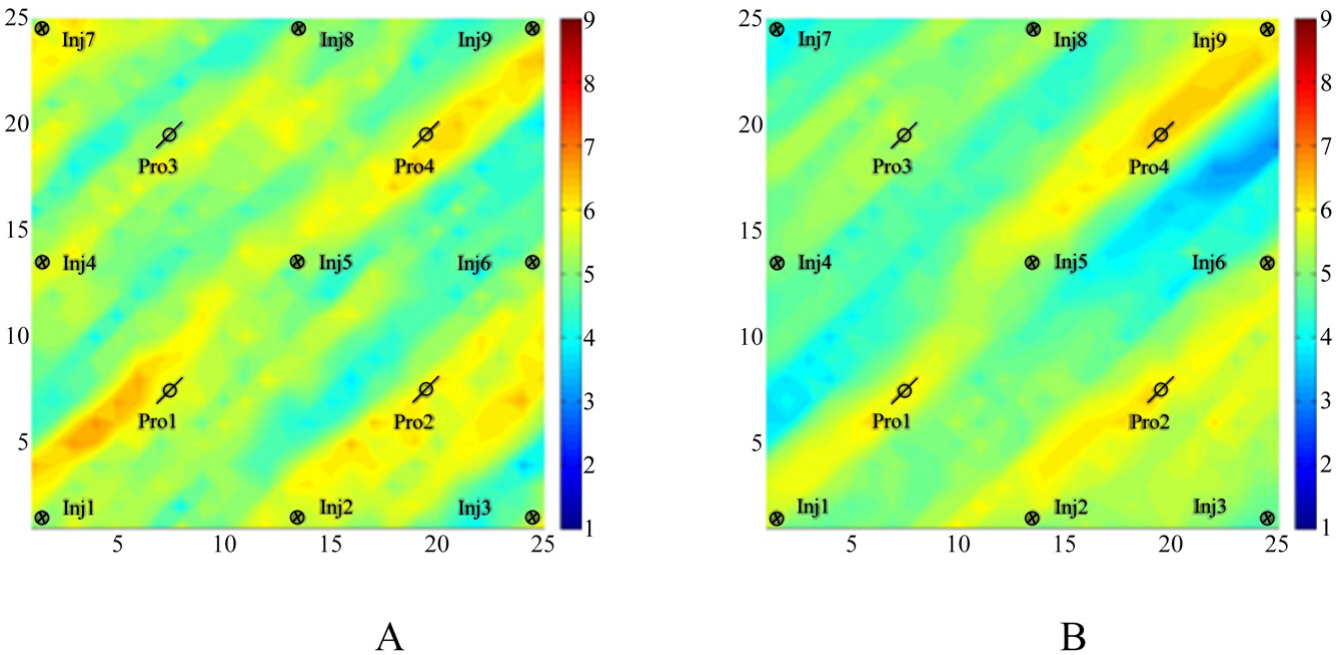
The matching result of the two algorithms. A:The simulation result obtained by Algorithm II, B: The simulation result obtained by Algorithm III.

From Fig 8, we can see that several hypertonic stripes appear in the matching permeability field, and the trends of the stripes are similar to those in the reference permeability field. In Fig 8(A), the distributions of the hypertonic stripes are clearly in the matching permeability field but are very different from those in the reference permeability field because of the randomness of the gradient obtained by using Algorithm II. In Fig 8(B), the distribution of the hypertonic stripes not only is clear in the matching permeability field but also is much closer to those in the reference permeability field. With this result, we can realize that by using Algorithm III, we can describe the reference permeability field more clearly than by using Algorithm II.

**Fig 9:**
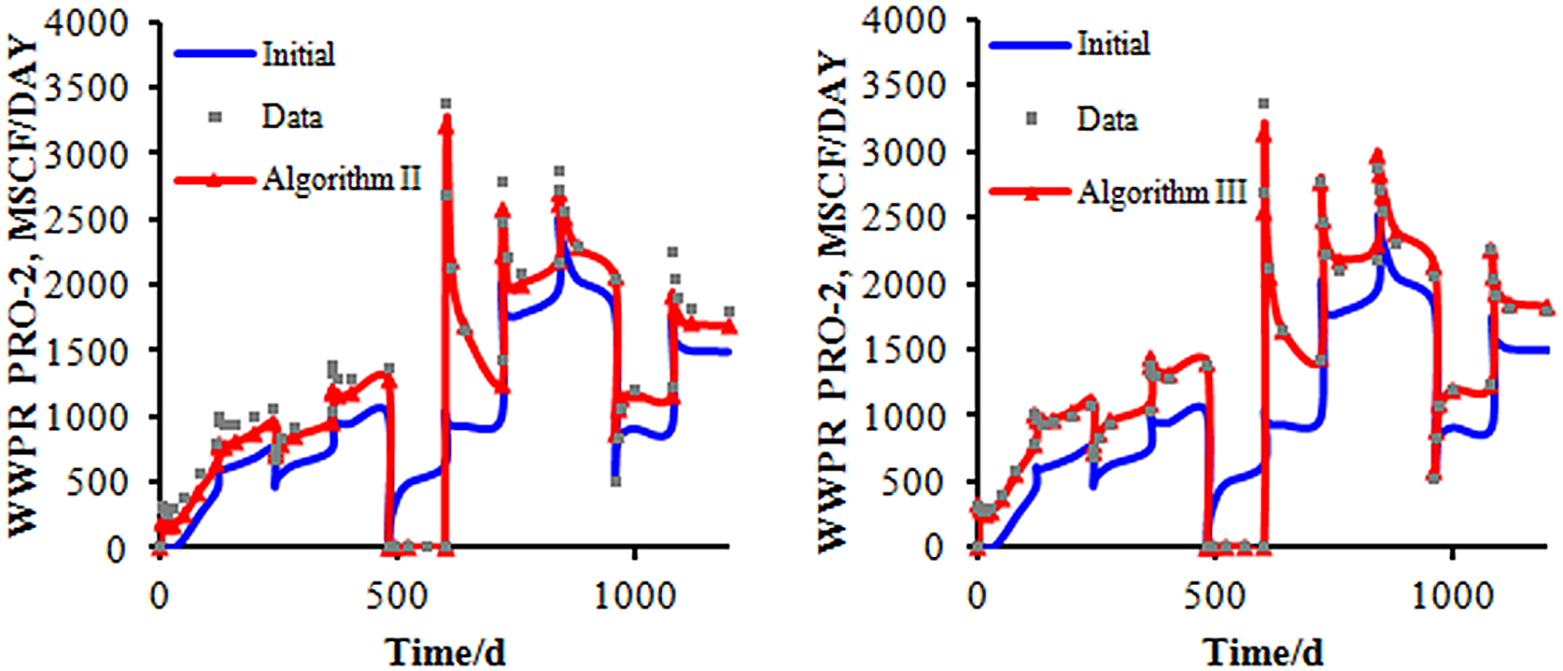
The matching result of the production data of the two algorithms. The bule line repesents the initial model’s simulation result; the gray points represent the observed data; the red line represents the final matching model’s simulation result.

**Fig 10:**
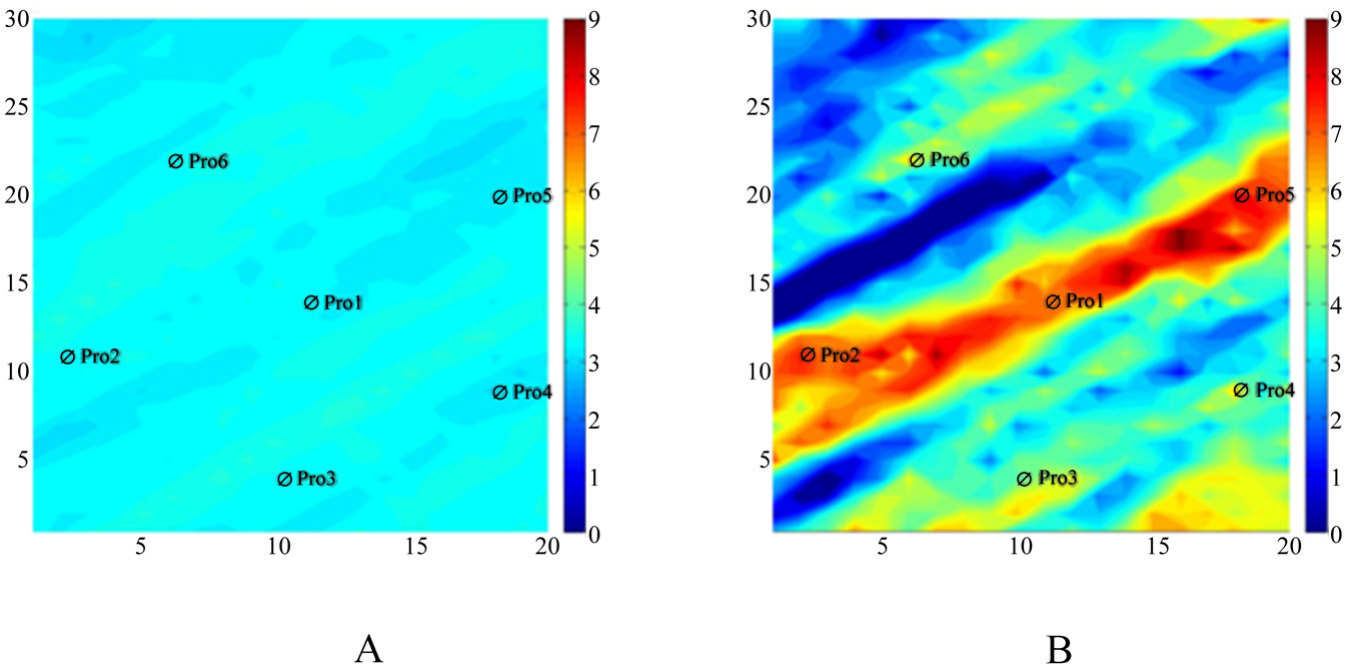
The different log-permeability distribution. A:The initial permeability field; B:the real permeability field.

Now, we analyze the optimization result of the production data by using both algorithms. Fig 9 shows the rate of water production of well Pro-4. The observed data varies greatly with the increase of time. We can obtain the simulation result that the stratigraphic parameters can be inversed by Eq 14. From Fig 10, we can realize that the matching curve from using Algorithm III is much closer to the observed data than that obtained by using Algorithm II.

### Case 2: Three-phase reservoir model

This reservoir simulation model is a 20×30 two-dimensional model, and it has a total of 6 production wells. In this model, the period of history matching is 9000 days, and every control step is 30 days. In other words, the well control parameters will be adjusted per every 30 days. In the process of history matching, the variables that must be resolved include the permeability at each cell, and the number of cells is 600. The production data that need matching include the bottom-hole flowing pressure, oil, gas and water production of wells.

In the production process of this reservoir model, the 6 wells began constant production, one-at-a-time in a sequence per 90 days. The first one is Pro-1, and the last one is Pro-6. After 1630 days, Pro-1 was converted into a water injection well while other production wells retained the same quota.

Before the start of the numerical simulation, we set the initial permeability field, which is based on reservoir information, as in Fig 10(A). Fig 10(B) shows the real permeability field. Then, we used two algorithms for optimization.

**Fig 11:**
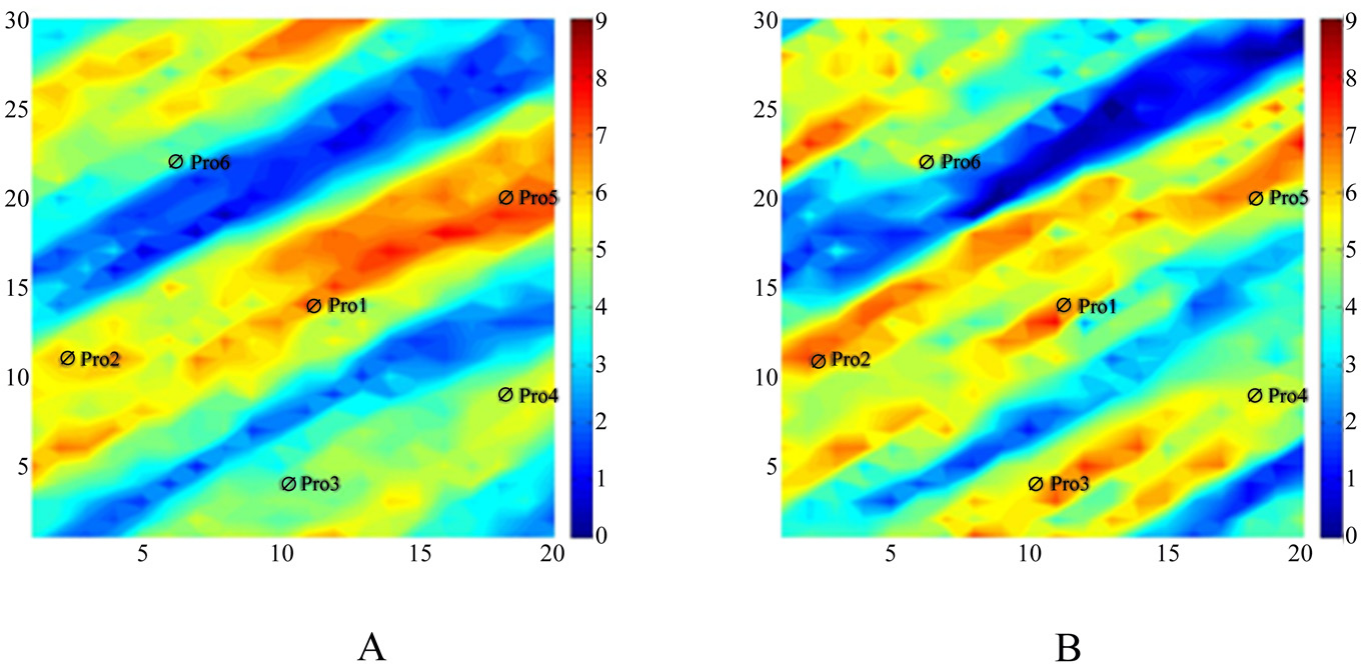
The matching results of the two algorithms. A:The simulation result obtained by Algorithm II, B: The simulation result obtained by Algorithm III.

Fig 11 shows the estimate of the log permeability that was obtained by history matching of the observed bottom pressure and the WOR (water oil ratio) data with the two algorithms. The matching result obtained by Algorithm II, as shown in Fig 11(A), shows the basic permeability channel structure without exactly matching the truth.

In Fig 11(B), the matching result shows that the matching result by Algorithm III has correctly reflected the permeability channel features, especially near the production wells where the reservoir characteristics can be exactly described.

**Fig 12:**
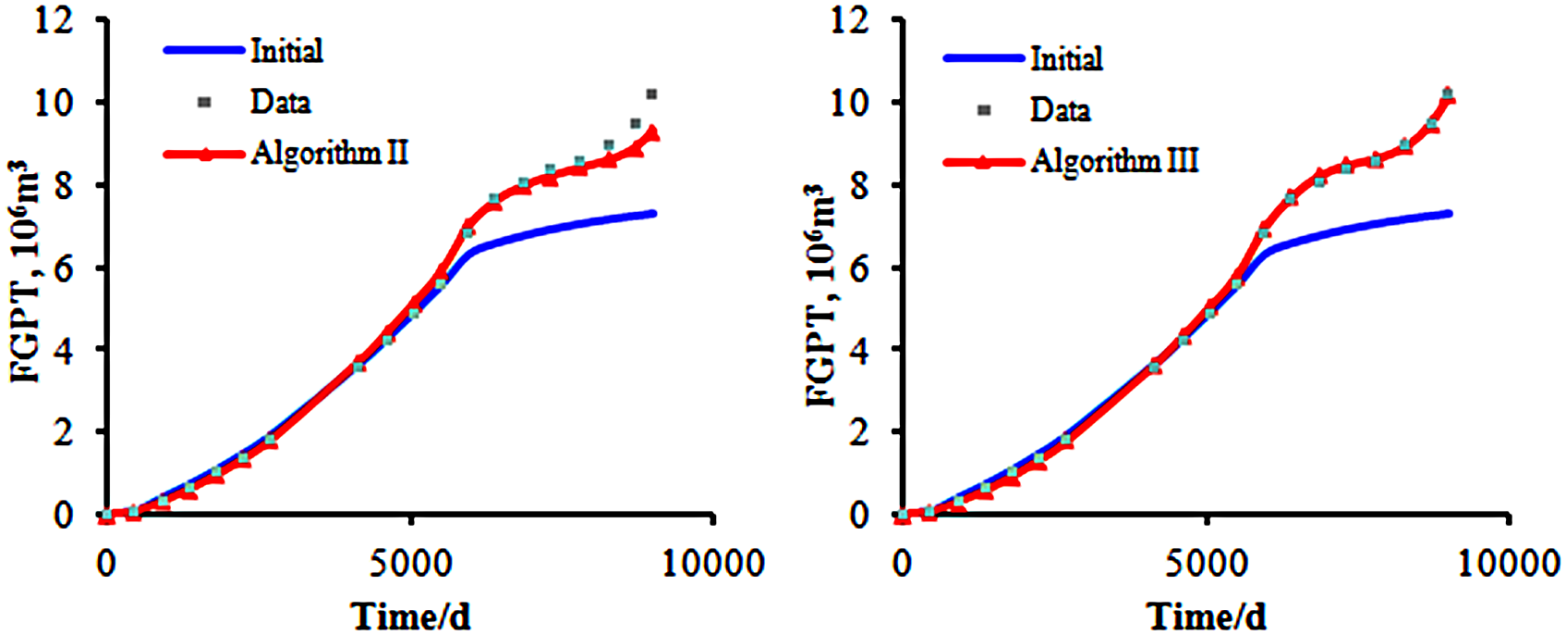
The total gas production of the two algorithms. The bule line repesents the initial model’s simulation result; the gray points represent the observed data; the red line represents the final matching model’s simulation result.

From Fig 12, we can see that there emerges a very large gap between the initial and observed data. After the history matching by using the two algorithms respectively, the results agree well with the observed data.

The matching results of the cumulative gas production are shown in Fig 12. In the early stages of oil field development, the gas production data of both the initial and real model are consistent. Those data begin to change at the 6000th day. After producing for 9000 days, the cumulative production of gas in the real model is 1.0203×10^7^ *m* ^3^. The matching result by Algorithm II is 9.3057×10^6^ *m* ^3^, while the matching result by Algorithm III is 1.0191×10^7^ *m* ^3^. Algorithm III has a better matching effect than Algorithm II.

**Fig 13:**
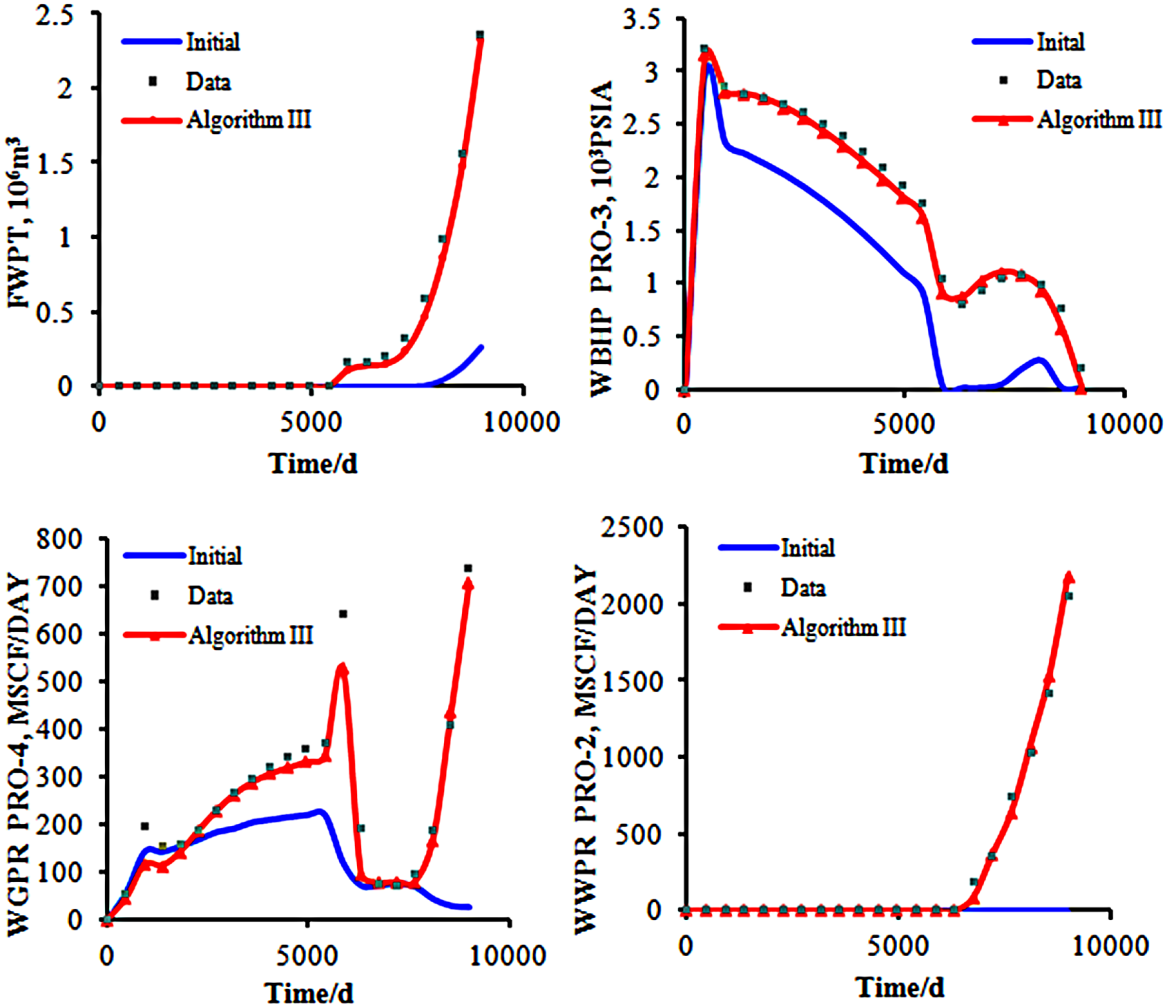
The matching result of the production data by Algorithm III. The bule line repesents the initial model’s simulation result; the gray points represent the observed data; the red line represents the final matching model’s simulation result.

Both Algorithms II and III have the same matching result for the other production data. Fig 13 shows the matching result of the other production data by Algorithm III. We can realize that through Algorithm III, we can obtain a satisfied optimization result, and Algorithm III can resolve the inverse problem of optimization.

**Fig 14:**
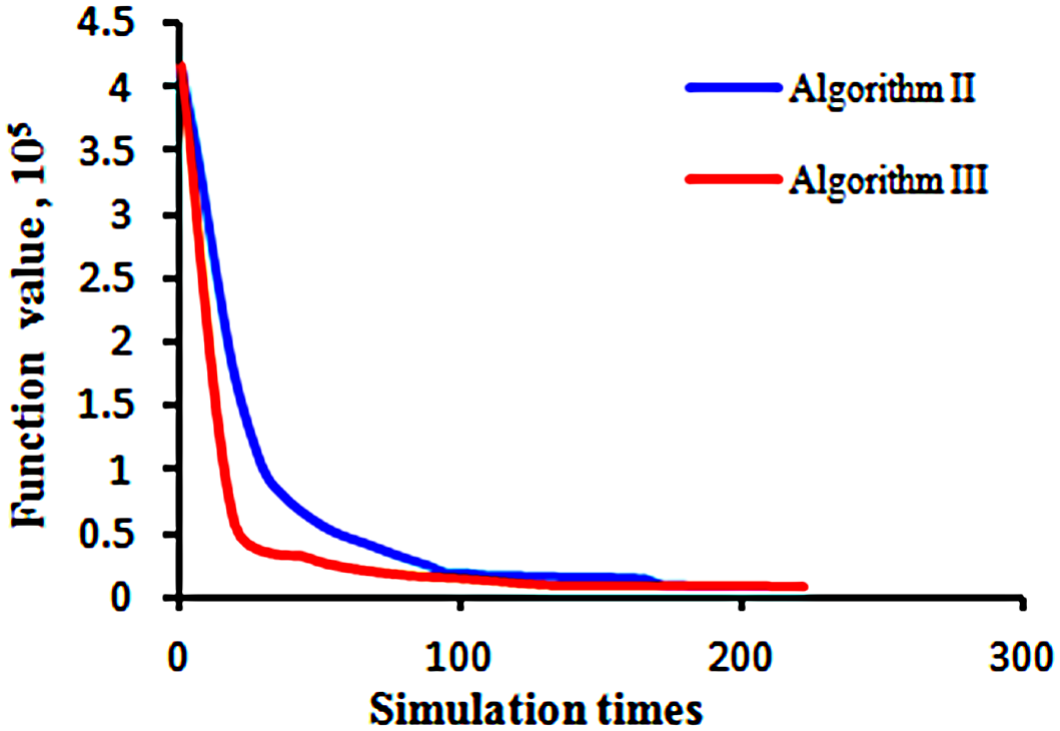
The convergence of the objective function. The blue line represents the objective function value versus the simulation runs by using Algorithm II. The red line represents the objective function value versus the simulation runs by using Algorithm III.

One of the criteria for comparing two algorithms is the rate of convergence of the objective function. The initial objective function value is 4.16×10^5^ (Fig 14). After a sufficient number of simulations, both of the algorithms could minimize the objective function value to near zero.

From Figs 12 and 13, we can require that both algorithms can match the production data very well. In Fig 14, we can see that the objective function optimized by Algorithm III has a higher speed of decline than that obtained from Algorithm II initially, but after 100 iterations, the objective function values of the two algorithms are nearly the same.

Through the above comparison of several aspects, we can see that Algorithm III has a higher rate of convergence and reflects the geological information more clearly than Algorithm II, but the variance matrix constituted by the formation parameters must be reduced dimensionally by decomposition before the optimization (in Problem Formulation part), which greatly reduces the number of parameters (it reduces to 100 in this paper). This method increases the errors between the optimization parameters and the observed data. Thus, when there are too many formation parameters, tthe advantages of Algorithm III over Algorithm II are not very obvious. Therefore, there exists potential for further research in this field.

## Conclusions

The present paper has investigated the application of finite differences with a stochastic algorithm for history matching in reservoir models. We use this method to optimize and resolve the Bayesian inverse problem, which depends on posterior distributions. Stochastic algorithms (such as SPSA, directional derivatives) determine the direction of the gradient with a random perturbation. The disadvantages are that the direction of the approximate gradient deviates from the direction of the true gradient; there are a large number of iterations; it has a slow rate of convergence, and it is vulnerable to local loops. The Hybrid algorithm obtains an approximate gradient that is much closer to true gradient by partial finite difference. It increases the accuracy of the search direction and improves the rate of convergence. This paper has verified (by the mathematical model and reservoir examples) that the Hybrid algorithm has the following advantages:

- Hybrid algorithm introduced with finite difference on the basis of a stochastic algorithm has greatly improved the accuracy of the approximate gradient, and this gradient is closer to the true gradient as the iteration steps increase;
- The approximate cosine formula to determine the accuracy of the approximate gradient has a high degree of accuracy, which provides a criterion to judge the accuracy of the approximate gradient for actual reservoirs;
- Compared with the stochastic algorithm based on a directional derivative, the Hybrid algorithm has a faster rate of convergence and can better describe geologicalinformation.
